# Establishment and recent surge in spatio-temporal spread of dengue in Nepal

**DOI:** 10.1080/22221751.2020.1740062

**Published:** 2020-03-23

**Authors:** Krishna Prasad Acharya, Bhim Chaulagain, Narayan Acharya, Kshitiz Shrestha, Supram Hosuru Subramanya

**Affiliations:** a Department of Livestock Services (DLS), Animal Quarantine Office, Kathmandu, Nepal; b Ministry of Land Management, Agriculture and Co-operatives (MoLMAC), Pokhara, Nepal; c Botany and Plant Pathology Department, Oregon State University, Corvallis, OR, USA; d Texas-Tech University, Lubbock, Texas, USA; e University of Melbourne, Melbourne, Australia; f Manipal College of Medical Sciences, Pokhara, Nepal

**Dear Editor,**

Nepal is vulnerable to the outbreak of many infectious diseases, including emerging and re-emerging diseases with significant impacts on ecosystem functioning and human health. Its rich biological diversity features three eco-climatic zones which are susceptible to invasive pathogens: tropical terai, subtropical and temperate regions in the mid-hills, and subalpine to alpine zones in the Himalaya [1,2]. Environmental degradation, changes in land-use patterns, agricultural intensification, and unplanned urbanization contribute to increased outbreaks of infectious diseases in Nepal. Moreover, low socioeconomic status, improper sanitation, poor public health facilities, and lack of awareness create a favourable situation for the emergence of and outbreaks of several infectious diseases including Japanese encephalitis (JE), malaria, and dengue fever in Nepal [3,4]. These diseases contribute significantly to socioeconomic burden in Nepal, with an increasing impact from dengue fever in recent years. Several studies on spatiotemporal epidemiology of this disease indicate that the situation is likely to worsen in the future due to climate change.

**Figure 1.**
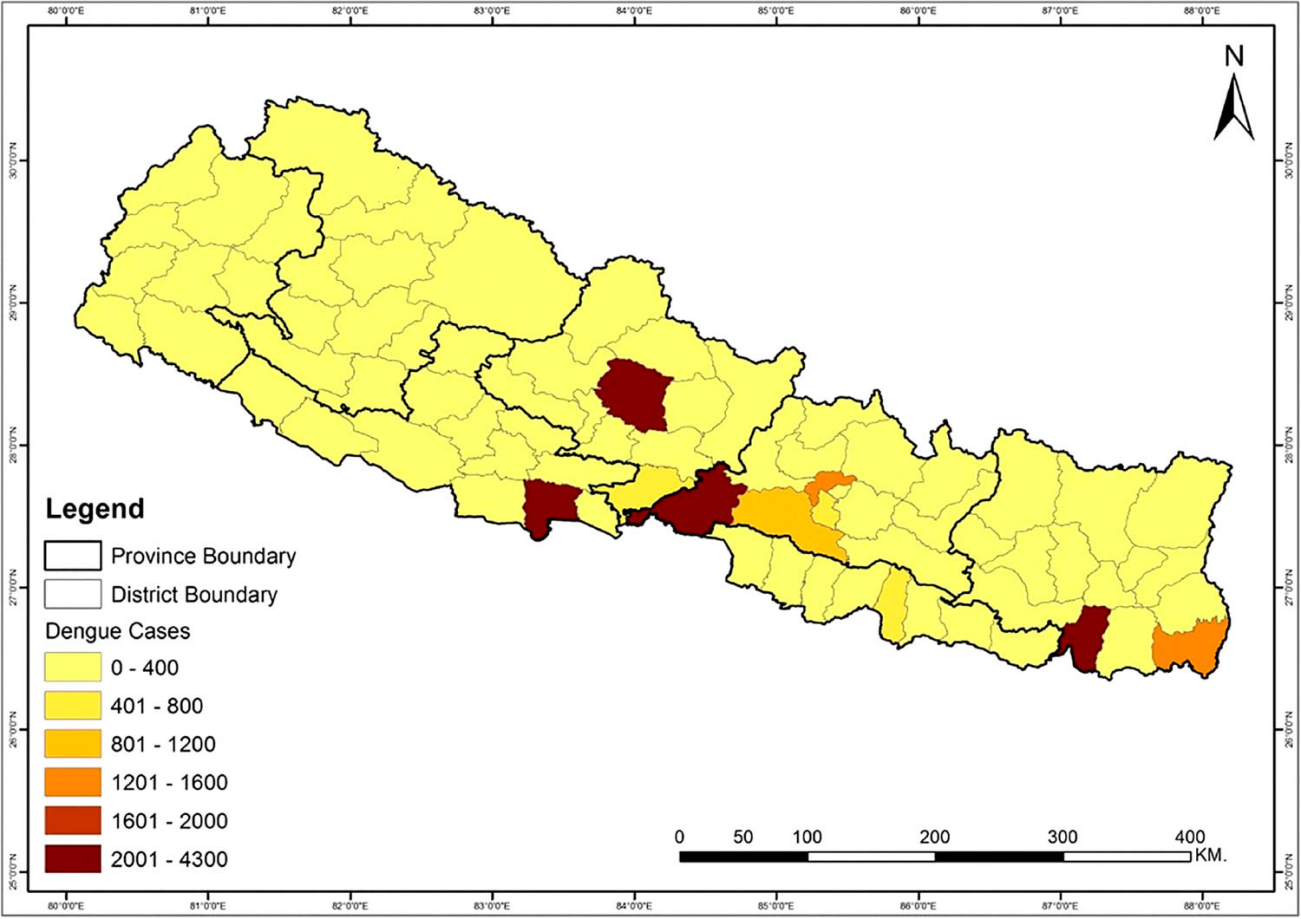
Map of the total number of confirmed dengue cases reported from different districts of Nepal from fiscal year 2014/15 until 2019/20. Data were obtained from the Epidemiology and Disease Control Division, Ministry of Health and Population, Government of Nepal.

**Figure 2.**
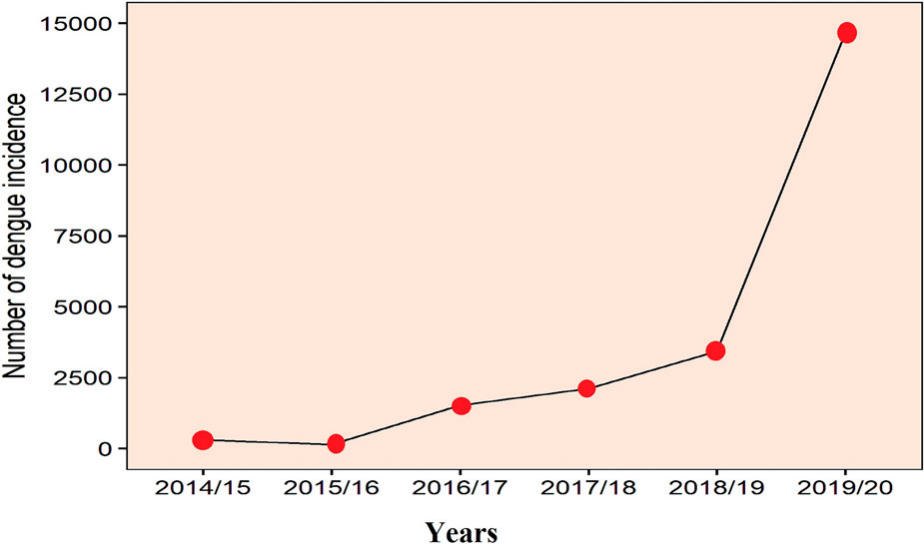
Graph representing the total number of confirmed dengue cases in each fiscal year starting from 2014/15 until 2019/20. Data were obtained from the Epidemiology and Disease Control Division, Ministry of Health and Population, Government of Nepal.

Dengue fever is an infectious viral disease from tropical and subtropical regions which is transmitted to humans from infected female mosquitoes, *Aedes aegypti* and *Aedes albopictus* [5]. Four dengue serotypes capable of causing infection have been identified so far (DENV-1, DENV-2, DENV-3 and DENV-4) [6,7]. A dramatic increase in global dengue fever in recent years has put half of the world’s population at risk, with an estimated 390 million people infected each year [5]. Since there is no specific treatment for dengue fever, it is now considered to be one of the biggest global health crises. Dengue fever was first reported in Nepal in 2004, and was followed by outbreaks that have been larger both in geographical area and in number of people infected [8]. This disease is now well established in the tropical and subtropical regions of Nepal and is migrating towards the hilly region of the country due to increasing temperatures associated with climate change. During the period of the first outbreak through 2014, only 2442 cases and five deaths were reported across 32 districts [9]. However, in the last five years, 21,858 confirmed cases have been reported across 60 districts including the mid-hills of Nepal (Figures 1 and 2). Large dengue outbreaks in Nepal occurred in 2010 (917 cases), 2013 (683 cases), and 2016 (1,527 cases) [2,10,11]. During these outbreaks, the major serotypes were identified as DENV-1 in 2010 and 2016, and DENV-2 in 2013 [2]. All four infectious serotypes of dengue virus (DENV-1–4) are present within the hosts, vectors, and the ecosystems of Nepal [12]. In 2019, the worst epidemic to date developed with 14,662 confirmed cases (Figure 2) [11], indicating a major shift in the temporal and spatial spread of dengue in Nepal. More than two serotypes are estimated to be involved in the current outbreak, and patients are exhibiting considerable differences in symptoms compared to previous years.

The risk of dengue infection and outbreak in Nepal is increasing year by year at different spatial scales [2,10,13]. Limitations of diagnostic tools and poor health care systems continue to be the major challenges in the early detection and diagnosis of dengue [2]. It is likely that the official tally of disease incidence is under-reported due to the complex nature of the disease and inadequate monitoring across the country. The majority of dengue patients are asymptomatic or present mild symptoms, and people are rarely tested due to high medical expenses and limited access to medical facilities in many regions of Nepal. Recent dengue epidemic outbreaks also exhibit unusual behaviour compared to the previous epidemics with an increased number of cases observed in the highlands of the Himalayan region in the temperate to subalpine climate [14,15]. This change is alarming and suggests that its range is likely expanding from tropical to temperate regions, which could be associated with climate change.

The Ministry of Health and Population (MoHP) of the Government of Nepal (GoN) has developed an Early Warning and Reporting System (EWARS) to warn about potential outbreaks of the disease. Signatures of the potential emergence of the current epidemic were detected about half a year ago on the first week of May 2019 with sporadic cases in the country [16]. Although government authorities were informed early about a potential epidemic outbreak, they failed to quickly implement counterstrategies including early warning to the public which led to an unusually long response time once the actual outbreak occured. Generally, the temporal progress of an dengue epidemic in Nepal intensifies over several months. Outbreaks following the monsoon season co-occur with high humidity and ambient temperatures which favour the replication, maturation, and transmission of dengue vectors [17]. Post-monsoon period with high rainfall and heavy flooding resulting conducive conditions for vector breeding with increased disease transmission efficiency is linked with higher morbidity and mortality unless stringent prevention and control measures are enforced. Though a rapid increase in dengue cases have been observed since 2010, and spatial and temporal disease projections are known, the government of Nepal is failing to respond in a timely manner with appropriate management strategies.

In response to the gravity of this situation, the MoHP of the GoN has recently declared “free testing and supportive medication for all dengue suspected patients” [18,19] and released a bulletin for “National Guidelines of Prevention, Control & Management of Dengue in Nepal 2019” in September 2019 [19]. Central, provincial, and local government levels have begun implementing prevention and control measures. For example, local authorities have started to destroy the breeding grounds of mosquitoes [20] and some provincial governments have declared free medical support to dengue patients [18]. People have been advised to participate in vector management through destroying potential mosquito breeding grounds including human-made stagnant water reservoirs, clearing bushy areas, utilizing fumigation, and avoiding mosquito bites by covering exposed skin and using mosquito nets or repellants [4]. Despite the limitations of these methods for destroying the vectors, these are good initiatives at different governmental levels to help manage the outbreaks of dengue fever.

Some independent research has been conducted to evaluate the spatial and temporal spread of dengue fever in Nepal [9,21]. These studies have provided the basis of understanding the factors that contribute to dengue incidence, epidemic outbreak, spread at different scales, and spatial and temporal clustering of disease. This information is very important for devising appropriate disease control strategies. Among several epidemiological parameters, the pathogen’s dispersal kernel most strongly influences the spread of the disease and the efficacy of control strategies. Since dengue fever exhibits long-distance dispersal (LDD) through its mosquito vector, the potential for it to rapidly spread over large spatial scales is greatly concerning. Traditionally, disease spread was modelled using a travelling wave approach with an epidemic front of constant velocity calculated using the pathogen’s reproductive capacity, generation time, dispersal ability, and vectors. However, disease spread for the pathogens exhibiting LDD is better characterized by fat-tailed dispersal kernels resulting in accelerating epidemic fronts [22]. Understanding these characteristics of epidemic spread is essential for the development of models that capture the large-scale processes that enable epidemiologists (or health care officials) to rapidly predict patterns such as rate of spread and the efficacy of potential outbreak interventions.

Some control measures for dengue fever have been effective in partially reducing the disease burden, yet these still fall below the level of control sought by the government as outlined in their Dengue Report [3]. This lag in control is due to a lack of trained professionals, inadequate monitoring and reporting systems, and insufficient designated departments authorized for carrying out large-scale control methods such as medical entomology. Therefore, the GoN should adopt stringent prevention and control measures by strengthening the disease surveillance system, upgrading the healthcare system with advanced facilities, and controlling the vector population to prevent and contain dengue epidemics. Additional research should be carried out to better understand the patterns of dengue outbreak, serotypes and genotypes involved, local and long-distance dispersal of mosquito vectors with the virus, spatial scaling of disease outbreak, and other epidemiological factors. Special attention should be paid to initial disease prevalence and spatial patterns, basic reproduction number of vector and the virus, and efficacy of control strategies. Environmental and demographic factors that influence dengue fever outbreaks should also be thoroughly considered while developing management strategies. All impacted sectors including every individual, household, and community should work proactively in conjunction with the government during periods of disease outbreaks at different temporal and spatial scales.
